# A Multi-Sensor Fusion Approach Based on PIR and Ultrasonic Sensors Installed on a Robot to Localise People in Indoor Environments

**DOI:** 10.3390/s23156963

**Published:** 2023-08-05

**Authors:** Ilaria Ciuffreda, Sara Casaccia, Gian Marco Revel

**Affiliations:** Department of Industrial Engineering and Mathematical Sciences, Polytechnic University of Marche, 60131 Ancona, Italy; s.casaccia@staff.univpm.it (S.C.); gm.revel@staff.univpm.it (G.M.R.)

**Keywords:** multi-sensor system, occupant localisation, PIR sensors, ultrasonic sensors, machine learning

## Abstract

This work illustrates an innovative localisation sensor network that uses multiple PIR and ultrasonic sensors installed on a mobile social robot to localise occupants in indoor environments. The system presented aims to measure movement direction and distance to reconstruct the movement of a person in an indoor environment by using sensor activation strategies and data processing techniques. The data collected are then analysed using both a supervised (Decision Tree) and an unsupervised (K-Means) machine learning algorithm to extract the direction and distance of occupant movement from the measurement system, respectively. Tests in a controlled environment have been conducted to assess the accuracy of the methodology when multiple PIR and ultrasonic sensor systems are used. In addition, a qualitative evaluation of the system’s ability to reconstruct the movement of the occupant has been performed. The system proposed can reconstruct the direction of an occupant with an accuracy of 70.7% and uncertainty in distance measurement of 6.7%.

## 1. Introduction

The ability to accurately determine the location of elderly people in their homes has the potential to support Ambient and Assisted Living (AAL) [1] scenarios in enhancing elderly people’s safety, health, and quality of life, as well as to enable the creation of more personalised and efficient care plans. However, despite significant advancements in computer vision and machine learning (ML) techniques, there are still some issues to be faced [2,3]. To begin with, the presence of furniture and appliances in indoor environments decreases the ability to distinguish between occupants and objects [4,5], creating an ambiguity problem. Moreover, people may not want their movements to be tracked, especially in their own homes, and ensuring privacy while still accurately localising occupants poses a significant challenge [6]. With regards to the former, the most common methods used to deal with the ambiguity problem in localising occupants in indoor environments are:

- Multi-Sensor Fusion approaches [5,6,7,8]: They integrate data from multiple sensors such as cameras [9,10,11,12], microphones [13], passive infrared motion sensors (PIR), LiDAR, and ultrasonic sensors to improve the accuracy of the localisation system. By using data from multiple sensors, the system can reduce the ambiguity in location estimation and provide more reliable and accurate results.
- Beacon-based approaches [14,15]: They use beacons, which emit radio waves to provide location information. By placing beacons in different locations within an indoor environment, the system can determine the location of a person based on the strength of the signal and proximity to the beacons. This kind of approach can provide high localisation accuracy but requires significant infrastructure investments.
- Wi-Fi-based approaches [16,17]: They use the signal strength of Wi-Fi access points to determine the location of a person. By using a database of Wi-Fi access points and their signal strengths, the system can estimate the location of a person with reasonable accuracy. However, this approach can be affected by signal interference and changes in the environment.

To reduce ambiguity in the estimation of a person’s location, the solutions above require the presence of a number of fixed anchor nodes and the use of AI algorithms, which, however, increase system complexity and costs. Moreover, the accuracy of these systems heavily relies on the optimal placement of anchor nodes, which can be difficult to achieve in large or intricate indoor spaces. In the study presented, a novel sensor network for occupant localisation was developed by integrating multiple PIR and ultrasonic sensors onto an existing sensor-controlled social robot with the aim to achieve complete coverage of a house using a single system, thus eliminating the need for an expensive and complex sensing infrastructure with multiple anchor nodes. In this study, localization refers to the capability of the robot to detect the presence of the occupant and determine the occupant’s position concerning the robot’s location. The main objectives of the localization procedure proposed are to identify whether the occupant passes by the robot or not, in which direction the occupant is moving, and the distance between the occupant and the robot. In this way, relevant information on the movement of the occupant, the environment in which the person is moving, and the person’s behaviour can be retrieved. The research was partially conducted within the framework of the “VITALITY” project, which utilises the Misty II mobile robot as a monitoring system for elderly people in an indoor environment. In the framework of the project, the localisation of the elderly is the first step to successfully monitoring them. Thus, the proposed multi-sensor system offers advantages such as ease of use, low cost, non-invasiveness, privacy preservation, robustness, and reliability, making it suitable for deployment in elderly people’s homes compared to existing systems. The study assumes a constant position and orientation of the robot during data acquisition for occupant localisation since the focus is on localisation rather than on robot navigation. To address the issue of ambiguity, the paper proposes an approach that combines PIR and ultrasonic sensors with a well-designed activation strategy and data processing procedure. PIR sensor data are processed using a decision tree (DT) algorithm to determine the direction of movement of an occupant in relation to the robot’s position in the room. Ultrasonic sensor data are processed using a K-Means clustering algorithm to differentiate between moving and stationary objects, thus making it possible to measure the occupant’s distance from the sensor. The effectiveness of the system was demonstrated in a controlled scenario by two tests, where the occupant was instructed to perform predefined movements at four different distances with a regular gait. The occupant was successfully localised, and his movements were reconstructed based on the robot’s fixed position. The authors believe that this approach has the potential to enhance healthcare interventions and provide valuable information about occupants’ health and well-being to relevant stakeholders. The early detection of changes in the behaviour of the elderly in the AAL scenario can enable healthcare providers to deliver targeted and effective interventions that promote healthy ageing and improve the overall quality of life. The paper is organised as follows: Section 2 offers a literature review on the implementation of sensing technologies for the purpose of localising occupants in indoor settings by using PIR and ultrasonic sensors. Section 3 details the sensors employed in constructing the localisation network as well as the approach adopted to collect data. In addition, the metrics used for data processing and the tests carried out to localise occupants within an indoor environment are illustrated. Section 4 presents the results of these tests, along with the accuracies of the sensor data. Section 5 offers a discussion of the results, while Section 6 concludes the paper by outlining the main findings.

## 2. Related Works 

Localising occupants in a home environment by using non-invasive sensors is the first step in monitoring their activities and detecting possible changes in their lifestyle. However, the variability of the physical layout of houses and the presence of obstacles can cause ambiguity problems that need to be addressed. As reported in Section 1, there are three main types of approaches to reducing ambiguity in a home environment: Multi-sensor fusion-based approaches, Wi-Fi-based approaches, and Beacon-based approaches. Multi-sensor fusion-based approaches use sensors such as RGB cameras and microphones to locate occupants in their homes. The main limitation of this approach lies in the fact that these sensors raise privacy issues as they are very invasive. In addition, hardware and implementation costs are quite high, which represents a further drawback to the use of this multi-sensor fusion technology [18,19]. Recently, LiDAR sensors have been widely used to localize people in an indoor environment. LiDAR are very accurate sensors, with a wide range and a fast acquisition rate. However, the main limitation of this technology is its use in complex environments. Real-world indoor environments can be filled with various objects, furniture, and obstacles of diverse shapes and sizes. LiDAR may have difficulties detecting people who are partially occluded or positioned in blind spots [20,21]. However, with the use of PIR sensors and ultrasonic sensors, it is possible to overcome these issues by providing a flexible and economical approach to indoor localisation. Wi-Fi-based technologies, instead, offer a cost-effective and scalable solution for indoor localisation, but their accuracy can be affected by factors such as signal interference, building layout, and the number of APs or beacons deployed. Similarly, the accuracy of beacon-based technologies is also influenced by various factors, including signal interference, building structure, and the number of beacons used. 

Thus, it can be concluded that, among the three types of solutions proposed, multi-sensor fusion approach technologies using PIR sensors and ultrasonic sensors are the most viable option for solving privacy and ambiguity issues while minimising infrastructural costs. In this Section, studies in the literature dealing with technologies based on both PIR and ultrasonic sensors that proved to be a promising solution for the purpose of localising occupants in their homes are presented. As in most of the works in the literature, in [22], an indoor localisation system that utilises ceiling-mounted PIR sensors is proposed. The system consists of two PIR modules and aims to evaluate the accuracy of system design in relation to the positioning of the structure. The results demonstrate that with two PIR modules, it is possible to simplify the system design and achieve better estimation accuracy compared to a single PIR module, obtaining an RMSE (Root-Mean Square Error) of 0.31. In [23], the authors propose a sensor node composed of five ceiling-mounted PIR sensors to detect and localise people. The system is tested with different deep learning methods and particle filter approaches, achieving distance errors of 0.4 m and 0.25 m, respectively. In [24], a network of four ceiling-mounted PIR sensors is proposed. The system uses particle filtering to eliminate incorrect occupancy state transition. The method proposed achieves an accuracy of 98.3%. Despite demonstrating the potential of using multiple PIR sensors for human indoor localisation, the system proposed is unable to provide a flexible and useful solution for decreasing infrastructure costs, since the sensors are mounted on the ceiling of a room. 

Ultrasonic sensors are widely used in activity recognition (fall detection) [25] and obstacle detection to help visually impaired and blind people navigate obstacles [26,27]. In [28], the authors integrated an ultrasonic sensor on an Arduino board to detect obstacles with the aim to help blind people move around in an indoor environment. The system alerts people when they are close to an object with an accuracy of 95.2%. In [29], the authors propose a system that uses multiple ultrasonic sensors and machine learning techniques to detect and estimate the distance of objects in real-time. The system consists of four ultrasonic sensors arranged in a circular pattern around a central point, with each sensor providing distance readings to an object in its field of view. The authors used a neural network-based approach to process sensor data and perform object detection and distance estimation. The system is tested in different environments, and the results show that it can accurately detect and estimate the distance of objects. In [30], the authors used a deep learning-based approach to process sensor data and estimate the distance of obstacles. The system is tested in different environments, and the results show that it can accurately estimate the distance of obstacles and enable their avoidance. Few works in the literature use this type of sensor to measure the distance of moving people. In [31], a trilateration-based technique is used to estimate the location of a person based on the time-of-flight measurements of ultrasonic signals. The system is tested in a real indoor environment and the results show a localisation accuracy within 1 m.

While, on the one hand, it can be observed that there are quite a few works in the literature that integrate multiple PIR sensors, on the other hand, the same cannot be said for works that integrate multiple ultrasonic sensors. Similarly, it can be noted that there is a lack of studies where these two types of sensors are combined and/or implemented. Therefore, localisation using PIR and ultrasonic sensors in indoor environments remains an open challenge to which researchers are trying to respond with different technologies and approaches. Since the aim of the study presented here is to create a system to be installed in the homes of elderly people, it is advisable to use non-wearable multi-sensor solutions that have high accuracy and low costs. Based on these requirements, a system of multiple PIR and ultrasonic sensors that can improve occupant localisation while minimising privacy issues, infrastructural costs, and ambiguity issues is proposed. 

## 3. Materials and Method 

### 3.1. Misty II Robot

Misty II is a social robot equipped with more than 25 sensors, including capacitive sensors, microphone arrays, bump sensors, time-of-flight sensors, and a structural core depth sensor containing an RGB camera. Moreover, the robot is designed as an open platform, which allows for further expansion and customisation. Misty II utilises its structural core depth sensor in conjunction with a processing system to perceive its surroundings and enable autonomous movement. The robot’s Simultaneous Localisation And Mapping (SLAM) module is used to reconstruct the environment and track the robot’s position in the global reference system, although the specific algorithms and methodologies used are not the focus of this study.

It is important to clarify that the robot’s primary task is not simply detecting the presence of occupants in a room. In fact, the robot’s original purpose involves other tasks, for example, monitoring their behaviour, which need to be carefully developed and executed. This work focuses on the localisation of occupants and thus it is crucial to ensure that any additional task performed by the robot, e.g., its self-localisation in the space and the map reconstruction of the environment, is accurately and effectively designed and implemented.

### 3.2. System Design and Sensor Integration

**Figure 1:**
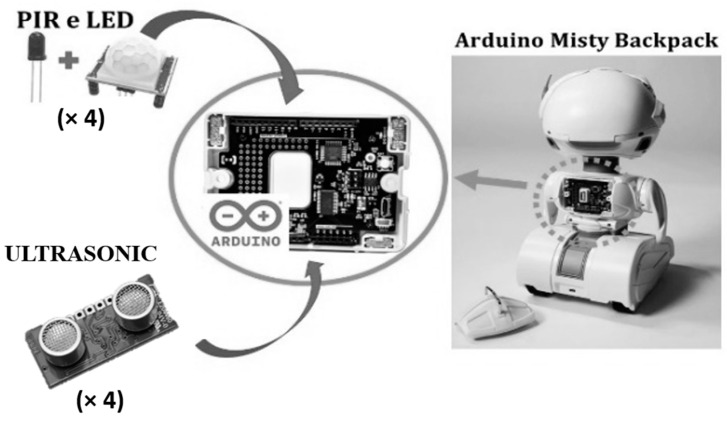
Integration of the HC-SR501 PIR sensors and the SRF10 ultrasonic sensors with the Arduino system of Misty II robot.

To detect and localise occupants using a stationary robot, a network of sensors needs to be installed to distinguish the occupants from their surroundings. The measurement system proposed aims to enhance the robot’s capabilities and is based on multiple sensors that were selected according to specific criteria, including cost-effectiveness, non-intrusiveness, and privacy considerations. Specifically, the sensors used to implement the system are HC-SR501 PIR sensors (refer to Table 1 for technical specifications) for motion detection, SRF10 ultrasonic sensors (refer to Table 2 for technical specifications) for distance detection, and an Arduino board (refer to Table 3 for technical specifications) mounted on the back of the Misty II robot (Figure 1), which serves as a power source and communication device with the database where data from the sensors are stored.

The PIR sensor is designed to detect changes in infrared radiation, specifically the heat emitted by living beings. When an occupant moves within the sensor’s field of view (FOV), their body heat causes a change in the infrared radiation detected by the sensor, which triggers the sensor to send a signal to the robot. The PIR sensor has three pins: VCC for power supply, GND for ground connection, and Out for signal transmission when movement is detected. The PIR sensors are connected to a breadboard for Arduino. The VCC and GND of the PIR sensors are wired with the positive pole and negative pole of the breadboard, respectively. The Out signal is connected directly to the general-purpose input/output (GPIO) Arduino board. The ultrasonic sensor emits a high-frequency sound wave, usually at 40 kHz, and then listens for the echo of that sound wave bouncing off an object. The time it takes for the echo to return is used to calculate the distance to the object using the speed of sound. This sensor has four pins: VCC for power supply, GND for ground connection, and two wires for the I2C bus protocol. As with the PIR sensors, the GND pin and VCC pin are connected to the negative and positive poles of the breadboard, while the I2C protocol uses two signal lines of the Arduino board: SDA (Serial Data) and SCL (Serial Clock). SDA is used for bi-directional data transfer between devices, while SCL is used for clock synchronisation between devices. In the study presented, to perform 360-degree localisation around Misty II and reduce possible ambiguity issues, a sensor network composed of 4 PIR sensors and 4 ultrasonic sensors was used. Each PIR sensor was paired with an ultrasonic sensor that was activated as soon as the PIR sensor was triggered. Considering the cost of each individual component, power supply, electronics, and storage, the system proposed costs approximately 300 euros, excluding the robot. The Misty II robot is Wi-Fi equipped, which makes it possible for it to be connected to the home Wi-Fi network. By taking advantage of this connection and installing sensors on the robot, it was therefore possible to transfer data from the robot to an external database service. Sensor data were sent to the database using robot application programming interfaces (APIs). To manage and manipulate data, a My Structured Query Language (MySQL) database was used. It is a fast, reliable, and scalable database solution that is widely used in web applications and software development that will be the basis for future system developments. It is worth noting that the system’s versatility allows it to be easily deployed on various robotic platforms, regardless of the type of robot used. 

### 3.3. Configuration of PIR and Ultrasonic Sensors

**Figure 2:**
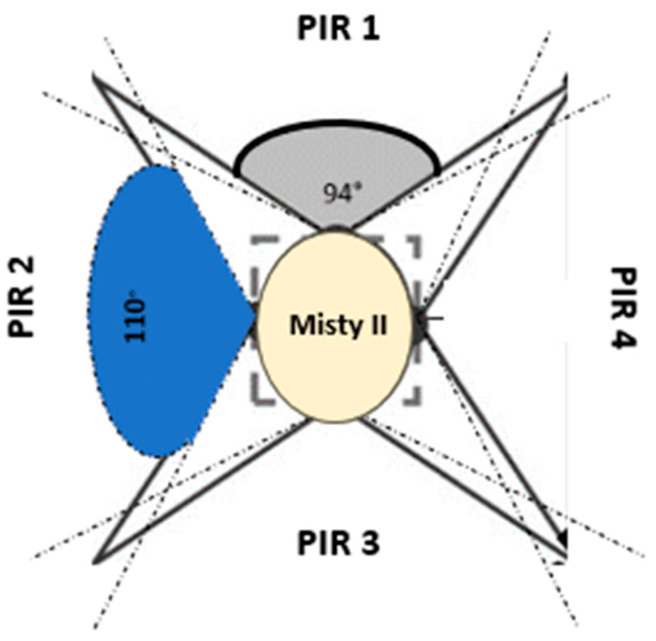
Arrangement of the PIR sensors on the robot’s head to achieve 360-degree coverage while minimising the area of overlap between adjacent sensors. The blue area corresponds to a FOV of 110°, while the grey area corresponds to a FOV of 94°. The x-axis represents the front of the robot, while the y-axis represents its left side.

**Figure 3:**
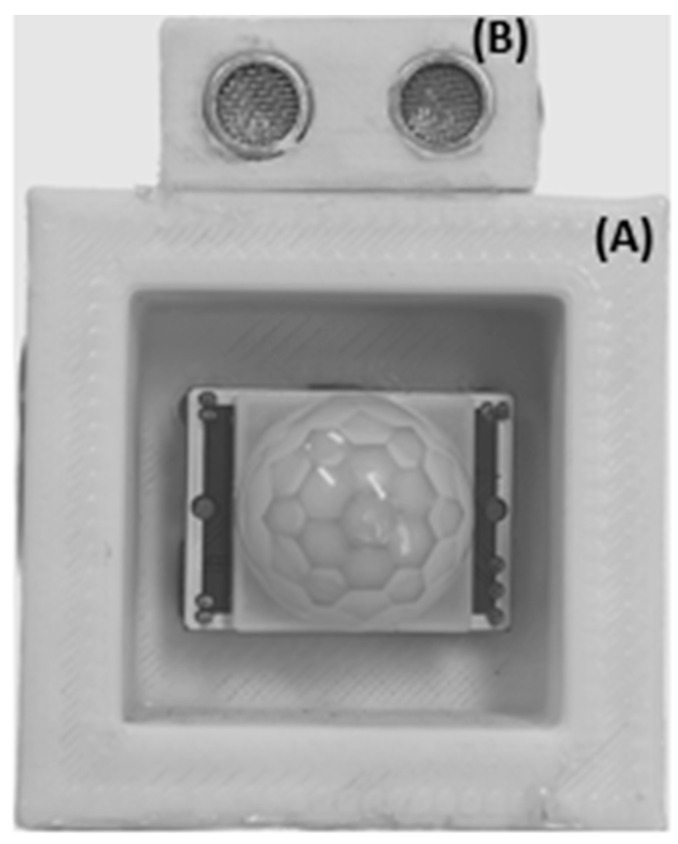
(**A**) is the box built for each PIR sensor to reduce its FOV. (**B**) is the box built for each ultrasonic sensor installed on top of the related PIR sensor.

In order to improve the detection accuracy of the PIR sensors, it is necessary to increase the number of sensors deployed. To achieve a 360-degree coverage area around the robot using PIR sensors with a FOV of 110° each, four sensors are required (110° × 4 = 440°). However, maintaining the same FOV for each sensor may result in a significant overlap of the FOV areas of adjacent sensors, leading to system saturation and difficulties in handling multiple activations of the PIR and the ultrasonic sensors. To address this issue, boxes were used to surround each PIR sensor, thereby reducing the overlap area between adjacent sensors from 110° to 94° (Figure 2). Additionally, a box was mounted above each PIR sensor to house the corresponding ultrasonic sensor (Figure 3). The four PIR sensors and four ultrasonic sensors were arranged in a square configuration on the robot’s head, with a side length of 14.5 cm.

This approach provides a solution to the problem of false-positive detections in PIR sensor systems and improves the accuracy and reliability of such systems in various applications, as explained in Section 3.5.1. The methodology proposed can be used in a range of settings, from security systems to robotics and automation.

### 3.4. Occupant Detection and Localisation

**Figure 4:**
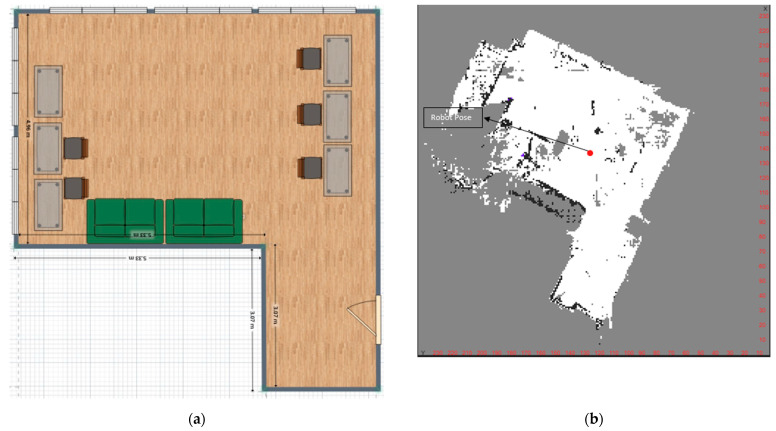
(**a**) Groundtruth map of the environment; (**b**) map reconstructed by the robot using the SLAM module. The representation of the environment is performed using different colours. The unknown areas are depicted in grey, the open areas in white, the occupied areas in black, and the coverage area in blue. The red dot indicates the robot Pose on the map.

An occupant tracking procedure that uses a mobile robot requires:

(a) A map of the environment in which the localisation is to be performed (Figure 4).
(b) The self-localisation of the robot in the environment.
(c) A system for localising people using the robot.

**Figure 5:**
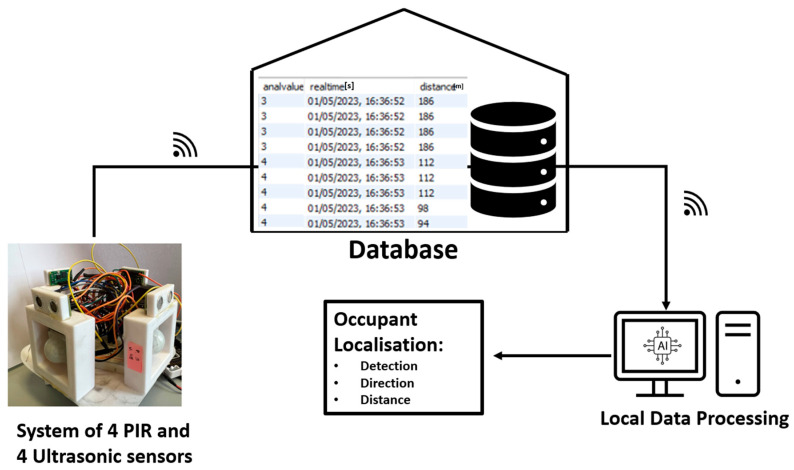
Occupant localisation setup.

It should be noted that the mapping of the home and the robot’s self-localisation are not included in the work, since they are considered to be beyond the scope of this study. Localisation, as intended in this study, through the sensor network proposed can be summarised in four steps:

(1) The 4 PIR sensors are constantly activated to detect occupant motion and direction.
(2) When a PIR identifies motion, the corresponding ultrasonic sensor is activated and identifies the distance of the moving occupant.
(3) The data on the active PIR sensor, acquisition time, and distance obtained from the ultrasonic sensor are stored in real time in the MySQL database (Figure 5).
(4) The data are then processed locally by an ML algorithm to extract information about the direction, detection, and distance, in order to localise the occupant in the environment considered (Figure 5).

### 3.5. Experimental Measurement Test Procedure

**Figure 6:**
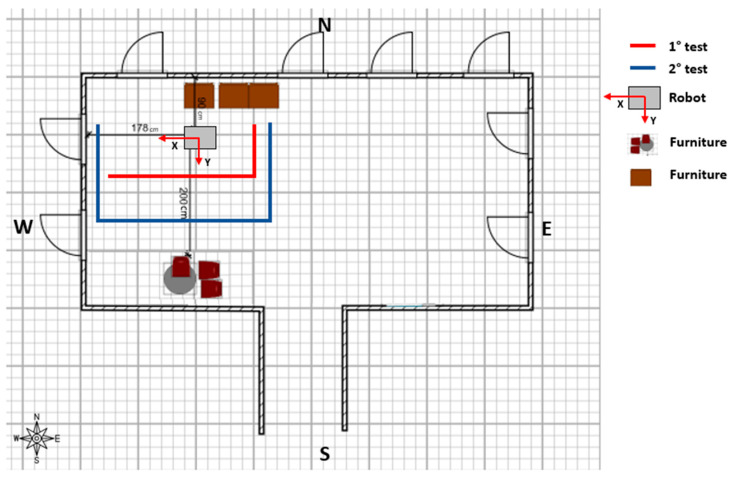
Floor plan of the scenario in which the tests were conducted. The red and blue lines indicate the first and second tests performed by the occupant, respectively. The robot is situated 178 cm away from the west wall and 90 cm away from the north wall. On the south wall, there is office furniture placed at a distance of 200 cm from the robot.

For the experimental measurement setup, the robot was equipped with a measurement system consisting of 4 PIR sensors and 4 ultrasonic sensors integrated with an Arduino board. Two tests were devised to assess the system in the indoor environment considered:

- The first test (red line in Figure 6) involved a movement comprising two linear segments at four different distances (0.5 m, 1.0 m, 1.5 m, and 2.0 m).
- The second test (blue line in Figure 6) involved a movement comprising three segments at four different distances (0.5 m, 1.0 m, 1.5 m, and 2.0 m).

During the tests, a subject was instructed to perform predefined movements at four different distances with a regular gait (Figure 6). The tests were conducted in an office room at the Department of Industrial Engineering and Mathematical Sciences of Università Politecnica delle Marche (Ancona, Italy), which contained furniture. Figure 6 illustrates the distances between the robot with the measurement system, the furniture, and the walls of the office room. The data were acquired at a frequency of 10 Hz then sent to the server and stored in the MySQL database to be post-processed to localise the occupant in the environment. At the end of the two tests, a database of 900 PIR activations with corresponding distance values was collected. 

#### 3.5.1. Occupant Detection and Direction Measurement

In this study, PIR sensors positioned on the head of a Misty II robot were used to accurately detect the direction of motion of an occupant using a decision tree (DT) classification algorithm. DT is a supervised machine learning algorithm used for both classification and regression tasks. The algorithm creates a tree-like model where each internal node represents a decision based on a feature, each branch represents the possible outcomes of that decision, and each leaf node represents the final predicted class or value [33]. Each PIR sensor was activated whenever the occupant was detected in its FOV. However, despite the reduction in the sensors’ FOVs using specially built boxes (Section 3.3), there was still some overlap with the FOV areas of the adjacent PIR sensors, which caused the adjacent sensors to activate, too. The DT aimed at distinguishing between the PIR sensors’ activations caused by the occupant’s movement and the false activations caused either by the overlap with the FOV areas of the adjacent PIR sensors or interfering sources such as glazed windows. It was also used to reconstruct the direction of the movement of the occupant. For these reasons, the DT was trained on models of PIR sensor activation to recognise the activation patterns corresponding to the occupant’s movement from those corresponding to false activations. The term “model” refers to the possible sequence of three consecutive activations of either one PIR sensor or two adjacent PIR sensors. These models were used to analyse the occupant’s movements and determine their location in the indoor environment in relation to the robot’s position. Considering the configuration of the PIR sensors reported in Figure 2, three examples of models used by the DT algorithm are shown to illustrate what happened when the occupant moved from PIR1 to PIR2.

**Figure 7:**
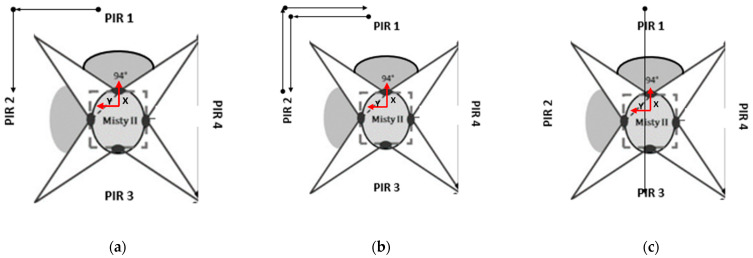
Examples of models used to train the DT: (**a**) The occupant moves from PIR1 to PIR2 and stays there for a certain period; (**b**) the occupant moves from PIR1 to PIR2 and then from PIR2 back to PIR1; (**c**) unauthorised movement because the occupant is unable to move past the robot.

In Figure 7a, the occupant moved from PIR1 to PIR2 and remained in PIR2. In this scenario, the activated PIR sensors were PIR1, PIR1, and PIR2, when the occupant moved within the overlapping area, and PIR2. In Figure 7b, the occupant moved from PIR1 to PIR2 and then returned to PIR1. In this scenario, the activated PIR sensors on the way from PIR 1 to PIR2 were PIR1, PIR1, and PIR2 when the occupant moved within the overlapping area, while on the way back from PIR2 to PIR1, the activated sensors were PIR2, PIR2, and PIR1 when the occupant moved within the overlapping area and PIR1. In Figure 7c, the occupant was incapable of moving past the robot, which resulted in an unauthorised activation sequence. 

A dataset of 45 models based on the three possible configurations described in Figure 6 was collected to train the DT. 

The DT algorithm was trained using 75% of the models generated, while the remaining 25% was used to test it. Accuracy (Equation (1)), precision (Equation (2)), recall (Equation (3)), and F1-score (Equation (4)) were computed to assess the performance of the DT algorithm.

(1) $$Accuracy\left[\%\right]=\left(\frac{\left(TP+TN\right)}{\left(TP+TN+FP+FN\right)}\right)*100$$

(2) $$Precision\left[\%\right]=\frac{TP}{TP+FP}*100$$

(3) $$Recall\left[\%\right]=\frac{TP}{TP+FN}*100$$

(4) $$F1score\left[\%\right]=\frac{2*PrecisionRecall}{Precision+Recall}*100$$

where TP = True Positive, TN = True Negative, FP = False Positive, and FN = False Negative. 

As reported in Section 3.5, the path of the first test was split into two segments, while the path of the second test was divided into three segments. Direction detection accuracy was considered as the ability to correctly reconstruct the direction of the entire movement covered by the occupant. It was computed on the two tests conducted in the controlled environment for the four distances using Equation (5).

(5) $$Accuracy\;[\%]=(\frac{number\;of\;correct\;segments}{number\;of\;total\;segment\;to\;be\;predict})*100$$

#### 3.5.2. Occupant Distance Measurement 

It is not possible to obtain information about the distance of the occupant from the PIR sensors. When the occupant is in its FOV, the PIR sensor activates and remains active, whether the position of the occupant changes or not. With the use of an ultrasonic sensor, instead, it is possible to measure the distance travelled by the moving occupant in time and create a movement profile. Specifically, when a PIR detects the presence of the occupant, it activates the ultrasonic sensor, which starts calculating the distance. It may happen that the occupant moves too fast and therefore the ultrasonic waves hit the walls of the room instead of the occupant, providing incorrect distance values. It is therefore necessary to use an algorithm that can discriminate the distances of fixed surfaces, such as room walls, from those that represent the occupant’s distances from the robot. For the study presented, the K-Means algorithm was selected. K-Means is a powerful unsupervised learning technique that can identify patterns in data, even in noisy environments. In fact, the k-means algorithm is used for clustering data into distinct groups or clusters based on similarity. It aims to partition the data into k clusters, where each data point belongs to the cluster whose mean (centroid) is closest to it.

When implementing the K-Means algorithm, it is necessary to:

1. Choose the number of clusters (k) that need to be identified. In the context presented, two clusters were necessary: One corresponding to the fixed objects, such as walls, and one corresponding to the occupant’s actual movements.
2. Select k random data points to serve as the initial centroids of the clusters.
3. Calculate the distance of each data point to each of the k centroids and assign the data point to the cluster corresponding to the nearest centroid.
4. Recalculate the centroids for each cluster as the mean of all the data points assigned to that cluster.
5. Repeat steps 3 and 4 until the centroids no longer change or until a maximum number of iterations is reached [34].

In the case presented, the K-Means algorithm gave two clusters: One representing the fixed surface and one representing the occupant’s movements.

Once the clusters are identified, this information is used to discriminate between the fixed surface and occupants’ movements based on the new distance measurements. In particular, each new measurement is assigned to the cluster with the nearest centroid. Over time, this information is used to determine whether the measurement corresponds to a fixed surface or an occupant when the sensor’s target is a fixed object such as a wall or a piece of furniture. Static ultrasonic tests have been found to achieve an error variation of approximately 3 cm. As a result, any centroid that exhibits movements smaller than 3 cm over time is categorised as a fixed point, whereas centroids that exhibit bigger movements are classified as representing occupants’ positions over time. The training of the algorithm is applicable whenever it is necessary to localise occupants in a home, as it is independent of the robot’s position. 

To estimate the measurement uncertainty of the distance values after the application of the K-means, the statistical confidence with a coverage factor of k = 2 is used, expressing the level of confidence that can be attributable to the measured value [35,36].

#### 3.5.3. Occupant Localisation 

**Figure 8:**
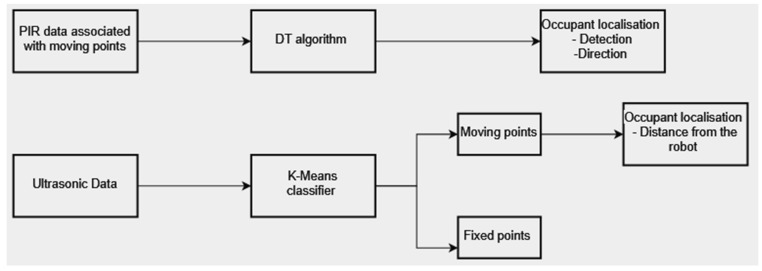
PIR and ultrasonic sensor data processing flow.

The data collected using the data acquisition methodology described in Section 3.4 were then transmitted and stored in the database. Local data processing started from the ultrasonic data with the aim to remove error data, e.g., data related to the walls. Thus, the ultrasonic data were first subjected to the K-Means algorithm to differentiate between the distances of moving and fixed points. Subsequently, the fixed points were used to filter out the data from the PIR sensors, resulting in the acquisition of PIR activation sequences associated with the distances of the moving points. The DT algorithm was then applied to the PIR sensor data to extract directional information regarding the occupant’s movement (Figure 8).

The reflection coordinates of the ultrasonic sensors were computed from each sensor perspective that detected the movement (Section 3.2). To this end, the Cartesian coordinates x_r and y_r of the reflection point (Equation (6)) were determined using the known location (x_s, y_s) and orientation (α) of the ultrasonic sensor, along with the measured distance (d) to the reflected object. In this context, x_r and y_r were assumed to be the origin of the Cartesian system. By applying the K-means algorithm, the points that were selected as the moving points were used to reconstruct the movement of the occupant inside the area of the corresponding PIR sensor.

x_r = x_s + d ∗ cos(α) (6)

y_r=y_s+d∗sin(α)

Through the integration of detection, direction, and distance information, it was possible to accurately determine the position of the occupant and reconstruct their movement.

## 4. Results

This section presents the results obtained from the experimental measurement procedure of the two tests by reporting the accuracies of the system in discriminating the direction and distance of the occupant’s movement from the robot.

### 4.1. Accuracy in Occupant Direction Measurement

Table 4 reports the precision, recall, accuracy, and F1-score values of the DT algorithm on the built templates, which were split into 75% for training and 25% for testing.

The system presented to detect occupant direction based on PIR sensors achieved accuracy in reconstructing the direction of the movement of the occupant of 70.7% and 66.3% for the first and second tests, respectively. The accuracy results for each distance in both tests are reported in Table 5.

### 4.2. Measurement Uncertainty in Occupant Distance Measurement

**Figure 9:**
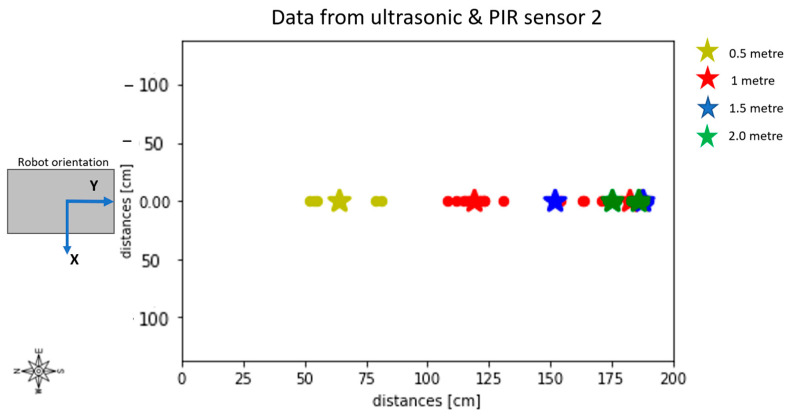
Movement of centroids obtained through the application of the K-Means classifier over time for PIR and ultrasonic Sensor 2 during the first test conducted at four different distances. The centroid positions at each of the evaluated distances are marked by stars, while the points linked with each centroid are represented by circles.

By applying the K-Means algorithm illustrated in Section 3.5.2 and studying the movement of the centroids over time, it was possible to distinguish between ultrasonic sensor data referring to stationary objects and those referring to moving objects. Figure 9 shows the results of the analysis conducted on the data collected from PIR 2 to study the movement of the centroids at different distances. As can be observed in Figure 9, at each distance, a centroid located close to 200 cm was identified. Referring to Figure 3, furniture was positioned at 200 cm from PIR 2. Therefore, all the points related to these centroid values were considered fixed points. The data points within each cluster of points classified as moving points were then utilised to calculate the measurement uncertainty expressed as the statistical confidence with a coverage factor of k = 2 for the four ultrasonic sensors in test 1 (8.5%) and test 2 (6.7%). 

### 4.3. Accuracy in Occupant Localisation Measurement

**Figure 10:**
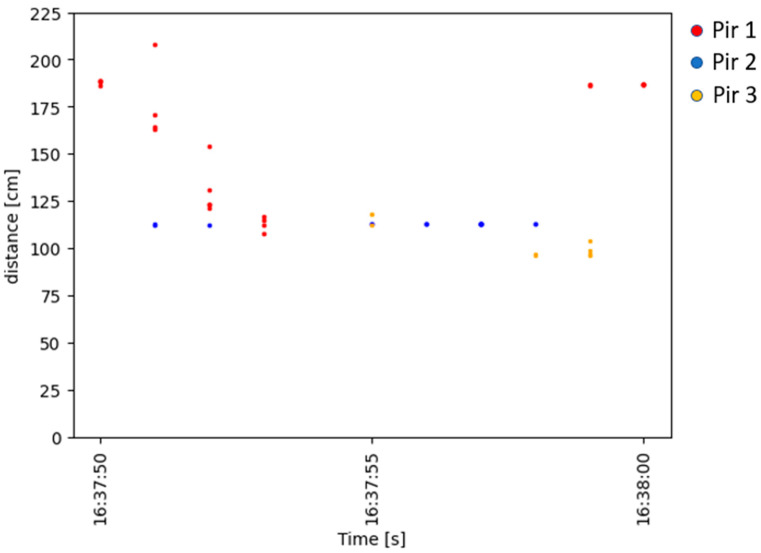
Data collected over time by the system composed of the four PIR sensors and four ultrasonic sensors.

**Figure 11:**
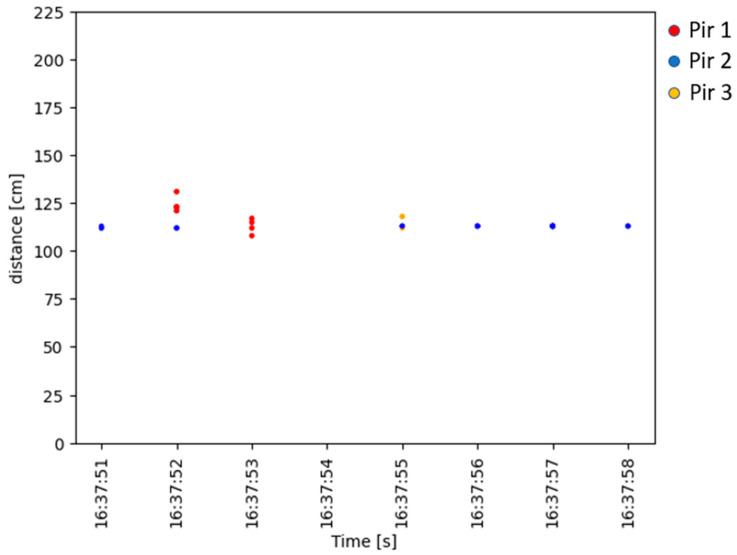
Data filtered over time by the K-Means algorithm to predict the distance of the occupant.

**Figure 12:**
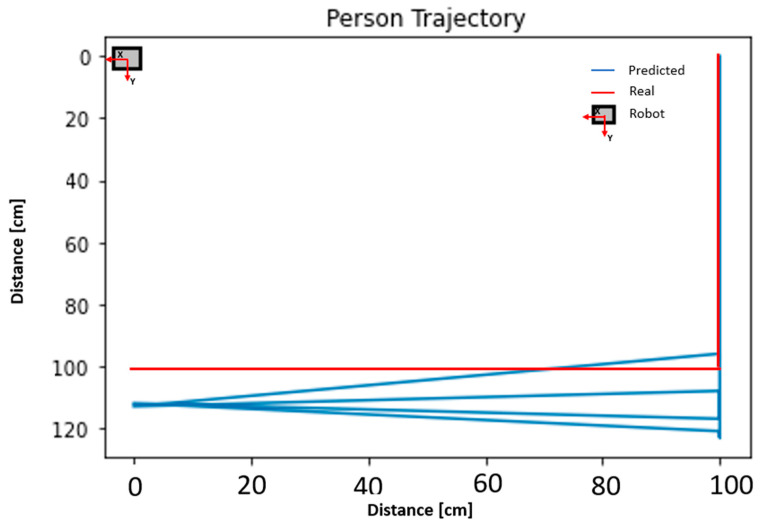
Occupant movement reconstruction based on DT and K- Mean classifier on the data collected from Test 1 at 1 m.

The system was designed to detect and localise an occupant in an indoor environment. The previous sections presented accuracy values for the PIR sensors and the measurement uncertainty expressed as the statistical confidence with a coverage factor of k = 2 for the ultrasonic sensors. In this section, an evaluation of the data obtained by applying the processing scheme described in Section 3.4. is presented. Figure 10 displays the data collected over time by the PIR and the ultrasonic sensors during the first test at a distance of 1 m from the robot. This figure shows how, for each activation of each PIR, a distance was recorded by the corresponding ultrasonic sensor. The multiple PIR activations per second depend on the acquisition frequency described in Section 3.5. By applying the K-Means algorithm to the ultrasonic data, it was possible to filter out the distances related to the fixed objects, which resulted in a significant reduction of data, as can be seen in the graph in Figure 11. The data related to the individual PIR activations obtained from this filtering process were then processed by the decision tree to extract information about the direction of the occupant’s movement. The reconstruction of the movement using the methodology proposed in this work is presented in Figure 12. The figure shows the difference between the predicted movement and the actual one with respect to the position of the robot located at the origin of the Cartesian axes.

## 5. Discussion

In this section, the importance of using multiple PIR and ultrasonic sensors on a robot to localise occupants in indoor environments is discussed. Moreover, we explored how the sensor activation and data processing techniques used in this study allowed for the measurement of the occupant’s movement direction and distance to reconstruct their movement.

According to the literature, most studies in this field focus on occupancy tracking and localisation strategies that use single-target sensors installed on ceilings. In this study, a novel system for the localisation and tracking of an occupant mounted on a mobile robot was presented. Compared to other approaches, the system proposed is easy to install, non-invasive for the person, respectful of privacy, and cost-effective as it makes it possible to reduce the infrastructure costs required for installing a sensor network in a house. Future research in this area will involve moving the robot to locate the occupant around the house. Moreover, unlike state-of-the-art systems, the configuration of the multi-sensor system presented provides 360-degree coverage around the robot, minimising the possibility of missing the detection of the occupant due to misalignment between the sensors and the occupant. In addition, the probability of having false-positive activations was decreased by building a box around the sensors to reduce their FOVs. These findings suggest that this type of system has the potential to revolutionise indoor localisation and tracking and could have practical applications in various fields, including safety and surveillance. Finally, the decision to activate the ultrasonic sensors only when the corresponding PIR sensors are active significantly reduced the amount of data to be processed. This approach has also made it possible to acquire synchronised data from the two sensors, avoiding additional data processing. 

In addition, to assess the robustness of the system, a qualitative analysis in reconstructing the occupant’s movement taking into account both distance values and the direction of occupant movement was conducted. Unsupervised (K-Means) and supervised (DT) machine learning algorithms were employed to process the data from the sensors. In this work, the DT trained on models built to discriminate the direction of the movement achieved an accuracy of 88%, a precision of 92%, a recall of 100%, and an F1 score of 88% (Table 4). The use of the two ML algorithms resulted in the successful localisation of the system proposed. The data flow diagram shown in Figure 8 illustrates the role of the two MLs in the filtering analysis to reduce the error in the reconstruction of the movement direction. Activating the PIR sensors on the distances of objects classified as fixed from the K-Means algorithm would have resulted in inconsistencies in the amount of PIR and ultrasonic data, losing synchronisation between the two sensors. Therefore, this approach can serve as a useful methodology for conducting preliminary data analysis for research studies. 

In Section 4.1, the authors presented the accuracy of the system in reconstructing the movement direction based on PIR sensors at distances ranging from 0.5 m to 2.0 m, with an intermediate step of 0.5 m. The results, which are shown in Table 4, indicate that, at a distance of 1 m, the system achieved 100% accuracy in predicting the direction of the occupant’s movement. However, as the distance from the PIR sensors increased (>1.5 m) or decreased (<2.0 m), the authors observed a corresponding decrease in the system’s ability to accurately reconstruct the direction of the occupant’s movement. These results provide valuable insights into the performance of the system since its ability to accurately locate an occupant in a house depends on the occupant’s distance from the system. If the occupant is too close or too far away, the system may not be able to effectively reconstruct the direction of their movement.

This condition may be due to the fact that, when the occupant walks too close to the sensor, the time spent in the FOV is very short. As a result, the system acquires very few data points, which are insufficient for the DT to accurately reconstruct the movement. Conversely, when the occupant walks too far away from the sensor, the stationary time in the sensor’s FOV is much longer. This produces a large amount of data that can cause the DT to malfunction, resulting in incorrect identification of the occupant’s direction.

Therefore, to optimise detection, it is essential to identify the optimal distance at which the robot should be positioned. Unfortunately, to the best of the authors’ knowledge, there is a lack of studies using similar systems in the literature, so a comparison of the accuracies is not available.

In Section 4.2, the measurement uncertainty for the two tests has been computed to evaluate the performances of the proposed system in the measurement of distances. The measurement uncertainty computed for test 2 (6.7%) is lower than the one of test 1 (8.5%), meaning that the proposed system exhibits robustness even in more complex paths or scenarios.

## 6. Conclusions

The main contribution of this work is the presentation of a multi-sensor system to localise an occupant in an indoor environment that uses four PIR sensors and four ultrasonic sensors installed on a mobile social robot. The advantage of this non-wearable system is that it is non-invasive, low-cost, and respectful of privacy. In addition, the system can be easily installed in a wide range of housing situations since it does not require infrastructural changes, unlike other systems proposed in the literature. Through tests carried out with an occupant, the work presented demonstrated that, by using the multiple-sensor system with the proposed sensor placement, it is possible to reconstruct the movement of an occupant in a home environment.

In addition, the analysis of the sensor data collected using both unsupervised and supervised machine learning algorithms proved to be an effective approach in increasing the accuracy of detecting and localising the occupant within the environment by determining their direction and distance of movement. This approach of combining different data from different sensors is accomplished using AI techniques since, through complex analysis, AI makes it possible to analyse heterogeneous data.

In future works, it is planned to conduct a real-time application to test the system in a real-life scenario where the robot and the occupant are free to move within a home environment. In addition, the DT and K-Means algorithms will be further investigated to increase the accuracy in localising occupants.

## Figures and Tables

**Table 1:** HC-SR501 PIR sensor technical specifications.

Sensor Specifications	PIR Sensor (HC-SR501)
Detection range	7 m
Detection angle	120°
Delay time	3 s
Consumption	65 mA
Operating voltage	4–12 V (5 V recommended)
Output voltage	3.3 V
Operating temperature	−15–70 °C
Cost	3 €

**Table 2:** SRF10 Ultrasonic sensor technical specifications.

Sensor Specifications	Ultrasonic Sensor (SRF10)
Detection range	43 mm–11 m
Frequency	40 kHz
Delay time	65 ms
Consumption	15 mV
Operating voltage	5 V
Connection	Standard I2C BUS [32]
Cost	25 €

**Table 3:** Arduino board technical specifications.

Specifications	Arduino
Microcontroller	ATmega328P
Operating Voltage	3.3 V
Input Voltage	7–12 V
Digital I/O Pins	14 (of which 6 provide PWM output)
Analog Input Pins	6
Flash Memory	32 kB
Clock Speed	8 MHz
Cost	25 €

**Table 4:** Percentage values of the accuracy, precision, recall, and F1-score of the DT algorithm trained by using the models for the possible occupant’s movement around the robot.

Algorithm	Precision [%]	Recall [%]	Accuracy [%]	F1-Score [%]
DT	92	100	88	88

**Table 5:** Percentage values of the accuracies of Test 1 and Test 2 at the four distance values computed using Equation (5).

PIRs to Occupant Distance [m]	Test	Accuracy [%]
0.5	1	33
	2	66
1.0	1	100
	2	100
1.5	1	100
	2	66
2.0	1	50
	2	33
Mean	1	70.7
	2	66.3

## Data Availability

Not applicable.
